# Responses of carbon, nitrogen, and phosphorus contents and stoichiometry in soil and fine roots to natural vegetation restoration in a tropical mountainous area, Southern China

**DOI:** 10.3389/fpls.2023.1181365

**Published:** 2023-05-09

**Authors:** Gang Hu, Zhonghua Zhang, Lei Li

**Affiliations:** ^1^Ministry of Education Key Laboratory for Ecology of Tropical Islands, College of Life Sciences, Hainan Normal University, Haikou, China; ^2^Key Laboratory of Wildlife Evolution and Conservation in Mountain Ecosystem of Guangxi, School of Environmental and Life Sciences, Nanning Normal University, Nanning, China

**Keywords:** C:N:P stoichiometry, tropical forest, vegetation restoration, fine root, nutrient limitation

## Abstract

The stoichiometry of key elements such as C, N, and P is an important indicator of ecosystem nutrient status and biogeochemical cycling. Nevertheless, the responses of soil and plant C:N:P stoichiometric characteristics to natural vegetation restoration remain poorly understood. In this study, we investigated C, N, and P contents and stoichiometry in soil and fine roots along vegetation restoration stages (grassland, shrubland, secondary forest, and primary forest) in a tropical mountainous area in southern China. We found that soil organic carbon, total N, C:P ratio, and N:P ratio significantly increased with vegetation restoration and significantly decreased with increasing soil depth, whereas there was no significant effect on soil total P and C:N ratio. Furthermore, vegetation restoration significantly increased the fine root N and P content and N:P ratio, whereas soil depth significantly decreased the fine root N content and increased the C:N ratio. The increasing average N:P ratio in fine roots from 17.59 to 21.45 suggested that P limitation increased with vegetation restoration. There were many significant correlations between C, N, and P contents and their ratios in soil and fine roots, indicating a reciprocal control of nutrient stoichiometric characteristics between them. These results contribute to our understanding of changes in soil and plant nutrient status and biogeochemical cycling during vegetation restoration and provide valuable information for restoration and management of tropical ecosystems.

## 1 Introduction

Eco-stoichiometry is a discipline that balances multiple chemical elements and energy flows in their ecological processes and interactions; it is a valuable tool for understanding biological processes from the scale of the organ to the ecosystem (Elser et al., 2000; Zhang et al., 2018). Carbon (C), nitrogen (N), and phosphorus (P) are the fundamental elements that make up living organisms and play a key role in the processes of energy flow and material cycling in the ecosystem (Sistla and Schimel, 2012). Soil and plant stoichiometry (i.e., the C:N:P ratio) has been broadly applied to infer biogeochemical cycling, nutrient limitation, and response to environmental change (Elser et al., 2010; Sardans et al., 2021). Ecosystem structure and function can be greatly altered by shifts in C:N:P stoichiometry induced by stressors such as vegetation degradation, land-use change, and atmospheric N deposition (Ning et al., 2021; Sardans et al., 2021).

The exchange of elements between soils and plants creates a mutual control of their elemental composition. Soil nutrient effectiveness directly affects nutrient uptake and assimilation by plants (Zhang Y. et al., 2019), whereas plants provide substrates for soil biology in the form of root and litter exudates, which drive C, N, and P cycling processes and thus affect soil nutrient effectiveness (Wang and Zheng, 2021). Therefore, soil C, N, and P contents and their stoichiometric ratios are important indicators of soil mineralization, nutrient cycling, and plant nutrient supply (Zhang W. et al., 2019). As an essential link between belowground and aboveground parts of the plant, fine roots (diameter ≤2 mm), which are the main organs for plant uptake, storage, and transport of water and nutrients, play a crucial role in energy flow and material exchange between plants and soil (Sarai et al., 2022). Consequently, the C:N:P stoichiometry of fine roots reflects the response and adaptability of plants to the environment, including the ability of C assimilation during plant growth and the efficiency of nutrient use and can be used to diagnose limiting elements (Nadelhoffer, 2000; Cao et al., 2020). There is a close connection between the plant organs and soil nutrients (Zhang et al., 2018; Dynarski et al., 2023). However, most previous studies have examined C:N:P stoichiometry in leaves (Lanuza et al., 2019; Li et al., 2021), and relatively little is known about root stoichiometry, especially for fine roots (Makita et al., 2011; Wang et al., 2020; Zou et al., 2021), presumably because it is more difficult to collect roots than leaves. Moreover, some current studies focused on the C:N:P stoichiometry of soils (Liu et al., 2022; Huang et al., 2023) or fine roots (Geng et al., 2022; Hu et al., 2022) and less on the nutrient relationships between soils and fine roots (Zhang et al., 2023).

Natural vegetation succession as a restoration mechanism is a common phenomenon in many human-disturbed sites around the world (Brus et al., 2020; Mudrák et al., 2021). Vegetation restoration is a synergetic process of plants and the soil environment that can improve nutrient cycling and soil quality of degraded ecosystems (Xu et al., 2019). The C, N, and P contents of ecosystem components (plant, litter, and soil) and their cycling patterns regulate plant survival and growth and various ecological processes in vegetation restoration (Wang and Zheng, 2021), and C:N:P stoichiometry reflects the feedback between vegetation dynamics and soil nutrients (Zhang et al., 2018). For example, Chen et al. (2018) revealed that fine root C:N:P stoichiometry changes with stand age of *Robinia pseudoacacia* and *Pinus tabulaeformis* forests and that the nutrient-use strategy differs between two temperate forest types as age increases. Similarly, Su et al. (2019) found that soil and root C:N:P stoichiometry showed a large response with vegetation succession in a high human-disturbance area in subtropical China. Recent few studies have indicated that soil and root C:N:P stoichiometry influenced by both vegetation type and soil depth (Chen M. et al., 2022; Joshi and Garkoti, 2023). However, our understanding regarding the synergy between the C, N, and P stoichiometric ratios of ecosystem components during vegetation restoration is still limited, especially in several important ecosystems such as tropical forests, which limits our grasp of the response of geochemical nutrient cycling to vegetation dynamics under the stressors of human activities and climate change.

Tropical forests, which are highly biologically complex but less understood ecosystems, play a critical role in protecting biodiversity, maintaining C and N balance, nutrient cycling, and regulating climate change (Wright, 2010; Mitchard, 2018). However, forest degradation and decline in tropics continue globally due to overexploitation, climate change, and land-use change, resulting in the loss of biodiversity and reduction in soil nutrients and carbon (Estoque et al., 2022; Smith et al., 2023). Therefore, restoration of tropical forest ecosystems is widely recognized as one of the most important and urgent challenges facing humanity and influencing climate change (Chazdon, 2019). Tropical soils are more vulnerable to inappropriate land management practices than temperate soils (Zhang et al., 2007). The degraded tropical soil undergoes rapid and irreversible chemical degradation through intense acidification, dissolution of aluminum, P fixation, depletion of cations, and reduction in organic matter (Lan et al., 2017). The majority of degraded forests worldwide are in the process of natural restoration, which aims to improve degraded soils while optimizing ecosystem structure and function (Verdone and Time, 2017).

The tropical mountain region in southern China is one of the world’s biodiversity hotspots (Myers et al., 2000). Dramatic population growth over the past century has increased demand for fuelwood, timber, and food, and intense human activities have destroyed forests with effects such as widespread red soil degradation and erosion, leading to biodiversity loss and forest fragmentation (Ren et al., 2007; Zhu, 2017). The vegetation in Shiwan Mountain in Guangxi Province of southern China is characterized by a typical tropical seasonal rainforest; however, the high human disturbance in recent decades has caused severe forest degradation and soil erosion in this area (Hou et al., 2010). To restore the ecological environment and make sustainable use of natural resources, China has implemented several ecological restoration projects nationwide since the 1970s, such as the Grain to Green Project and the Natural Forest Protection Project (Zhang et al., 2015). In this context, natural vegetation restoration after disturbance represents a general change of land use in southern China (He et al., 2011; Wang et al., 2018). Although the tropical mountains are extremely important providers of ecosystem services, little research has been carried out on the ecological processes of vegetation restoration on Shiwan Mountain. Only a few studies have reported that vegetation restoration significantly improved soil enzyme activity and soil microbial biomass in this tropical mountain area (Sun et al., 2014; Sun et al., 2015). However, it remains unclear how soil and plant stoichiometry respond to natural vegetation restoration in tropical China.

In the present study, we investigated soil and fine root stoichiometry at four vegetation restoration stages on Shiwan Mountain. The main objectives of this study were to (1) explore the shifts of C, N, and P contents and their stoichiometric characteristics in soil and fine roots along natural vegetation restoration in a tropical mountain area and (2) clarify the relationships between soil and fine root stoichiometric characteristics. To the best of our knowledge, this study is the first to analyze the soil and fine root stoichiometric characteristics along vegetation restoration in tropical China.

## 2 Materials and methods

### 2.1 Study area

**Figure 1:**
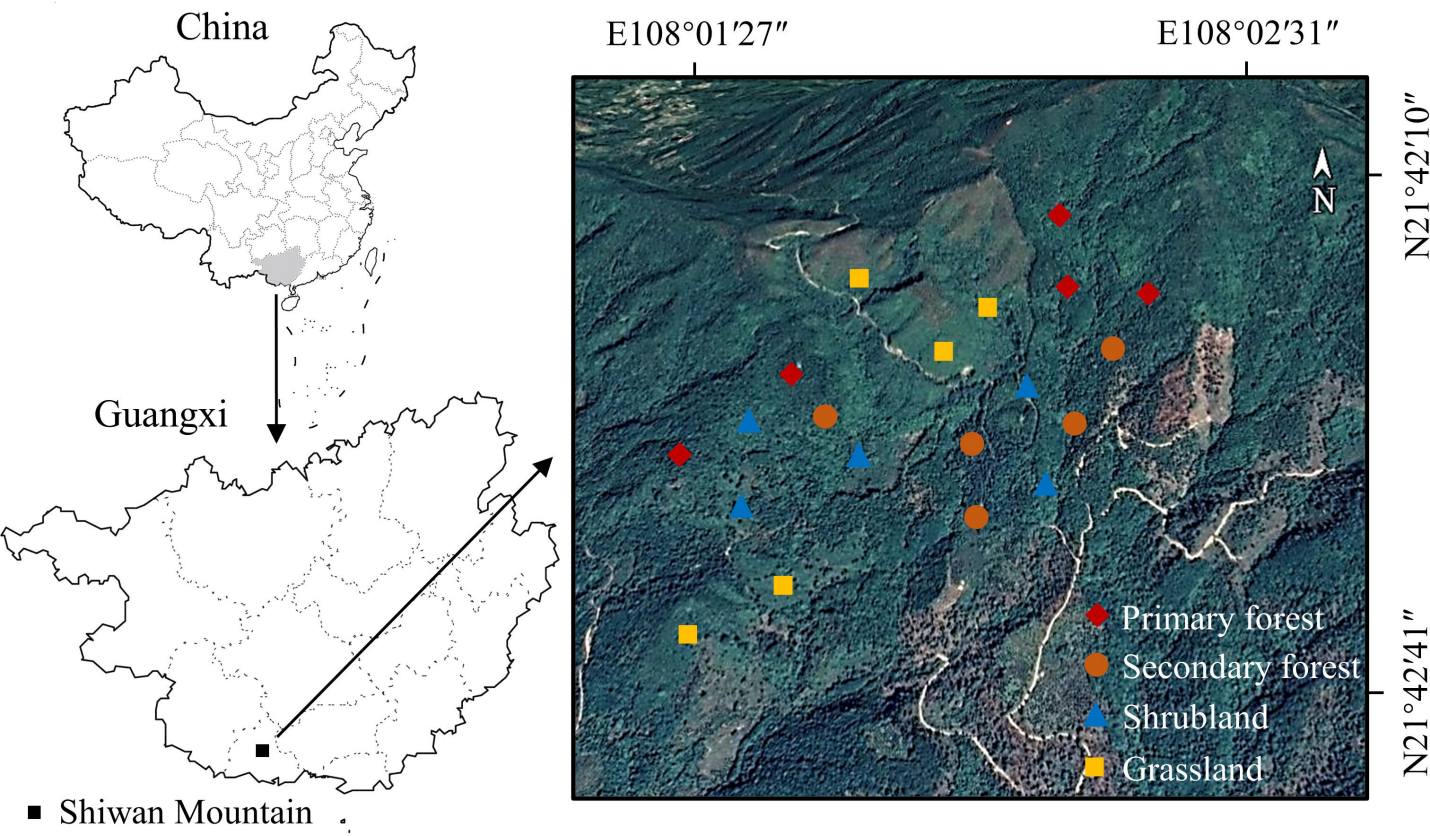
Sampling sites on a hillside in a tropical mountainous area in Guangxi Province, southern China.

The study area is located on Shiwan Mountain (108°02′E, 21°42′N) in Shangsi County, Guangxi Province, southern China (**Figure 1**). The study area belongs to the northern tropical monsoon climate zone with annual sunshine of 1,525 h, annual average precipitation of more than 2,900 mm, and an annual average temperature of 21.9°C (Sun et al., 2014). The soil types are red soil and brick-red soil, and the main soil-forming parent rocks are granite, sandstone, and shale. This mountainous area is characterized by a tropical seasonal rainforest, but due to long-term human disturbance, the primary forest has been highly fragmented (Sun et al., 2015). Most of the vegetation types in the sampling sites are secondary forest, shrubland, and grassland. **Table 1** describes the four vegetation types in detail.

**Table 1:** Information on sampling sites at four vegetation restoration stages in tropical China.

Restoration stages	Recovery age (a)	Elevation (m)	Slope (°)	Canopy cover (%)	Vegetation height (m)	Dominant species
Grassland	5–10	335–541	25.3 ± 1.3	87.4 ± 2.3	0.7 ± 0.1	*Eragrostis pilosa*, *Arundinella hirta*, *Eulalia speciosa*, *Dicranopteris dichotoma*, *Bothriochloa bladhii*
Shrubland	15–20	403–466	27.5 ± 2.1	77.4 ± 3.4	2.9 ± 0.5	*Rhodomyrtus tomentosa*, *Paeonia delavayi*, *Maesa japonica*, *Rubus alceifolius*, *Urena lobata*
Secondary forest	30–50	381–479	30.2 ± 1.2	84.4 ± 3.7	9.6 ± 0.8	*Madhuca pasquieri*, *Mytilaria laosensis*, *Schefflera octophylla*, *Archidendron eberhardtii*, *Diospyros morrisiana*
Primary forest	>100	424–576	26.6 ± 1.7	85.4 ± 4.3	16.5 ± 1.3	*Archidendron eberhardtii*, *Wendlandia uvariifolia*, *Ormosia pachycarpa*, *Eberhardtia aurata*, *Semiliquidambar cathayensis*

Values are the mean ± standard error.

### 2.2 Experimental design

During the peak growing season (August) in summer 2021, four vegetation restoration stages, namely, grassland, shrubland, secondary forest, and primary forest, were selected on a hillside on Shiwan Mountain (**Figure 1**) based on the spatio-temporal substitution method (Lu et al., 2022; Wu et al., 2022). Five plots, each of 20 m × 20 m, were established for each stage. The distance between each plot ranged from 50 to 470 m. These plots had similar environmental conditions, such as mean elevation, slope degree, and soil type. Vegetation surveys were conducted within each plot, and the species name, coverage, abundance, and height were recorded.

In each plot, five representative sampling points were selected according to the “S” shape. Soil and fine root samples were collected at three depths of 0–10 cm, 10–20 cm, and 20–40 cm using soil augers with an inner diameter of 10 cm, for a total of 60 samples (4 stages × 5 plots × 3 depths = 60). The samples of the same soil layer from the same plot were evenly combined to obtain a mixed sample for each of the three depths per plot, and the samples were transported to the laboratory. In the laboratory, fine roots (diameter ≤2 mm) in the soil cores were removed with tweezers and dried at 65°C to constant weight for 48 h. The dried fine roots were ground in a ball mill. The soil samples were air-dried in the shade and passed through a sieve with 2-mm mesh size. The soil organic carbon (SOC), soil total nitrogen (TN), and fine root total C and N were measured using a C and N analyzer (Vario MAX, Elementar, Germany). Total phosphorus (TP) of soil and fine roots was measured with a continuous flow analyzer (San ^++^, Skalar, Netherlands) after digestion with H_2_SO_4_–H_2_O_2_.

### 2.3 Statistical analysis

The effects of restoration stage, soil depth, and their interactions on C, N, and P contents and their stoichiometric ratios in soil and fine roots were examined by linear mixed models (LMMs). In the model, restoration stage, soil depth, and their interactions were considered as fixed factors, while plot term was treated as a random factor. One-way analysis of variance (ANOVA) with least significant difference (LSD) test was carried out to analyze significant differences among restoration stages and soil depths. The relationships between C, N, and P contents and their ratios in soil and fine roots were analyzed using Pearson correlation analyses. The analysis of LMMs and ANOVA were performed with SPSS version 21.0 (SPSS Inc., Chicago, IL, USA), and correlation analysis were conducted using the PerformanceAnalytics R package (Peterson and Carl, 2020).

## 3 Results

### 3.1 Variation of soil C, N, and P contents and their stoichiometry at four restoration stages

**Figure 2:**
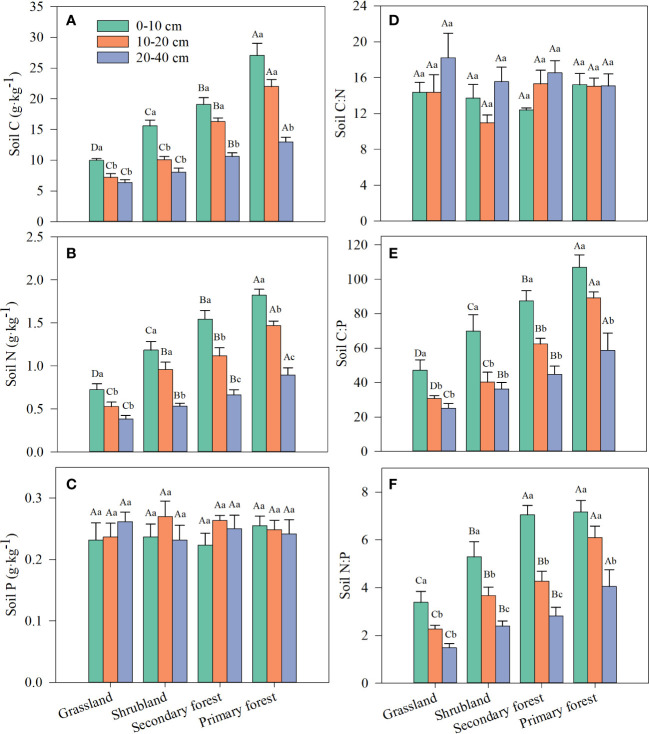
Changes in SOC **(A)**, TN **(B)**, TP **(C)** and stoichiometric ratios of soil C:N **(D)**, C:P **(E)** and N:P **(F)** along four stages of vegetation restoration. Different capital letters above the bars indicate significant differences among restoration stages, and lowercase letters indicate significant differences among soil layers (*p* < 0.05). Values are the mean ± standard error.

Vegetation restoration stage, soil depth, and their interaction had a significant effect on SOC and TN contents but not on TP content and C:N ratio (**Table 2**). Meanwhile, restoration stage and soil depth had a significant effect on soil C:P and N:P ratios, but their interaction did not (**Table 2**). SOC and TN contents and soil C:P and N:P ratios increased with restoration and decreased with increasing soil depth, and the highest contents and ratios were in the topsoil (0–10 cm) in primary forest, whereas the lowest were in the subsoil (20–40 cm) in grassland (**Figures 2A, B, E, F**; **Supplementary Table S1**). Soil TP content and C:N ratio did not vary significantly with restoration stage and soil depth (**Figures 2C, D**).

**Table 2:** Results from linear mixed models to evaluate the effects of restoration stage, soil depth, and their interaction on C, N, and P contents and their stoichiometry in soil and fine roots.

	Variables	Restoration stage (R)	Soil depth (S)	R × S
Soil	C	108.144^**^	83.897^**^	6.783^**^
	N	72.922^**^	93.232^**^	3.576^**^
	P	0.030	0.755	0.650
	C:N	1.225	2.344	1.047
	C:P	40.620^**^	39.113^**^	1.542
	N:P	33.090^**^	49.202^**^	1.610
Fine root	C	1.360	0.396	1.478
	N	79.146^**^	25.221^**^	1.362
	P	4.442^**^	0.805	0.170
	C:N	86.140^**^	19.600^**^	1.272
	C:P	5.490^**^	0.943	0.315
	N:P	3.394^*^	2.061	0.773

The F-values are shown in the table. ^*^p < 0.05, ^**^p < 0.01.

### 3.2 Variation of fine root C, N, and P contents and their stoichiometry at four restoration stages

**Figure 3:**
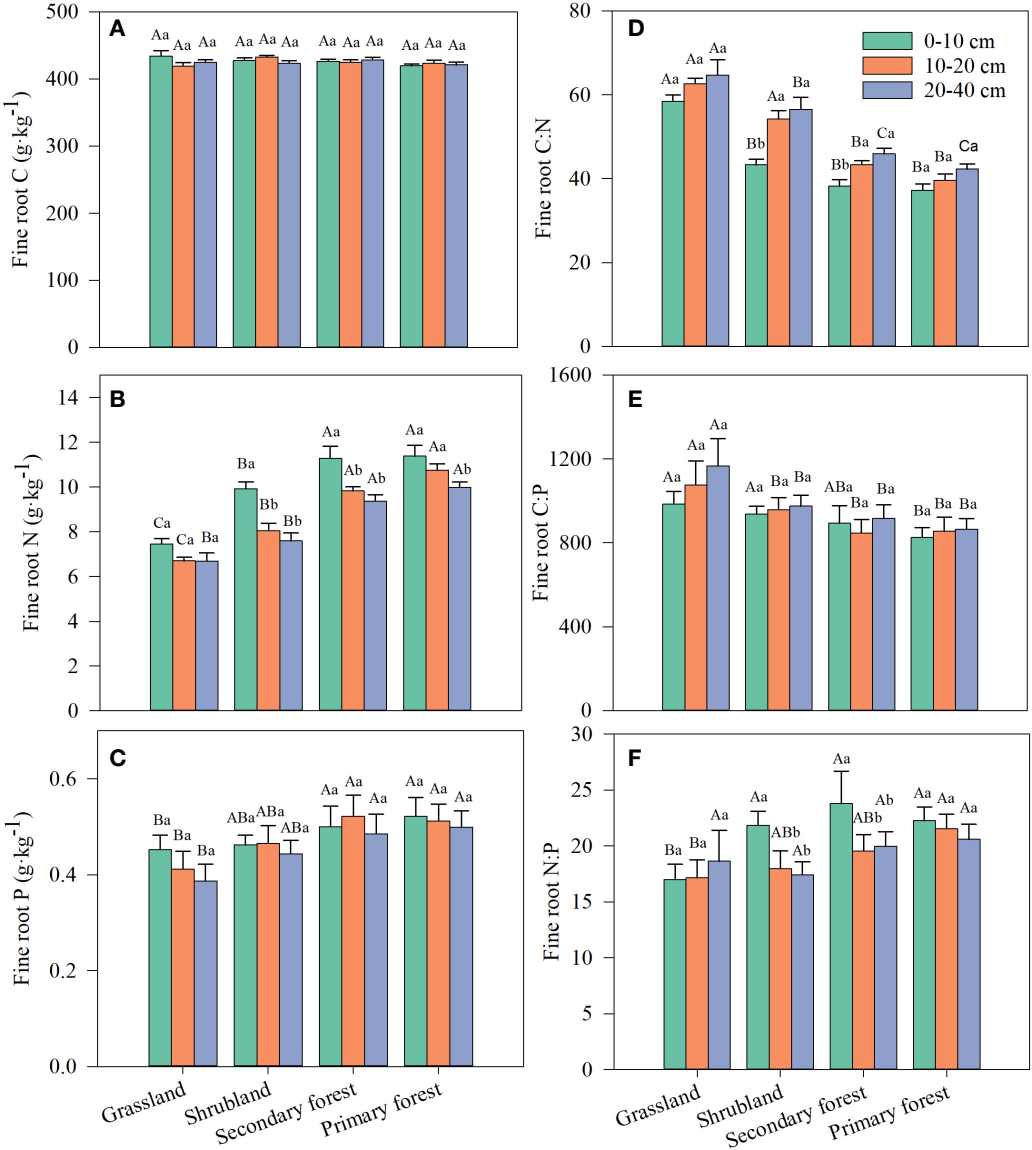
Changes in fine root C **(A)**, N **(B)**, P **(C)** and stoichiometric ratios of fine root C:N **(D)**, C:P **(E)** and N:P **(F)** along four stages of vegetation restoration. Different capital letters above the bars indicate significant differences among restoration stages, and lowercase letters indicate significant differences among soil depth (*p* < 0.05). Values are the mean ± standard error.

Fine root C content was not affected by restoration stage, soil depth, and their interaction, whereas fine root N content and C:N ratio were significantly affected by restoration stage and soil depth but not by their interaction (**Table 2**). Fine root P content, C:P ratio, and the N:P ratio only differed significantly with restoration stage (**Table 2**). Fine root N content increased with restoration and decreased with soil depth (**Figure 3B**; **Supplementary Table S1**). Fine root P content tended to increase with restoration but did not differ significantly with soil depth (**Table 2**; **Figure 3C**). Fine root P content at the three soil depths was significantly lower in grassland than in secondary and primary forest but did not differ significantly from that in shrubland (**Figure 3C**). Both fine root C:N and C:P ratios decreased with restoration in the same soil layer (**Figures 3D, E**), whereas fine root N:P ratio tended to increase with restoration (**Figure 3F**). Fine root C:N and C:P ratios increased or tended to increase with increasing soil depth, but fine root N:P ratio decreased the most with increasing depth in the four restoration stages (**Figures 3D–F**; **Supplementary Table S1**).

### 3.3 Relationships among the soil and fine root C, N, and P contents and their stoichiometry

**Figure 4:**
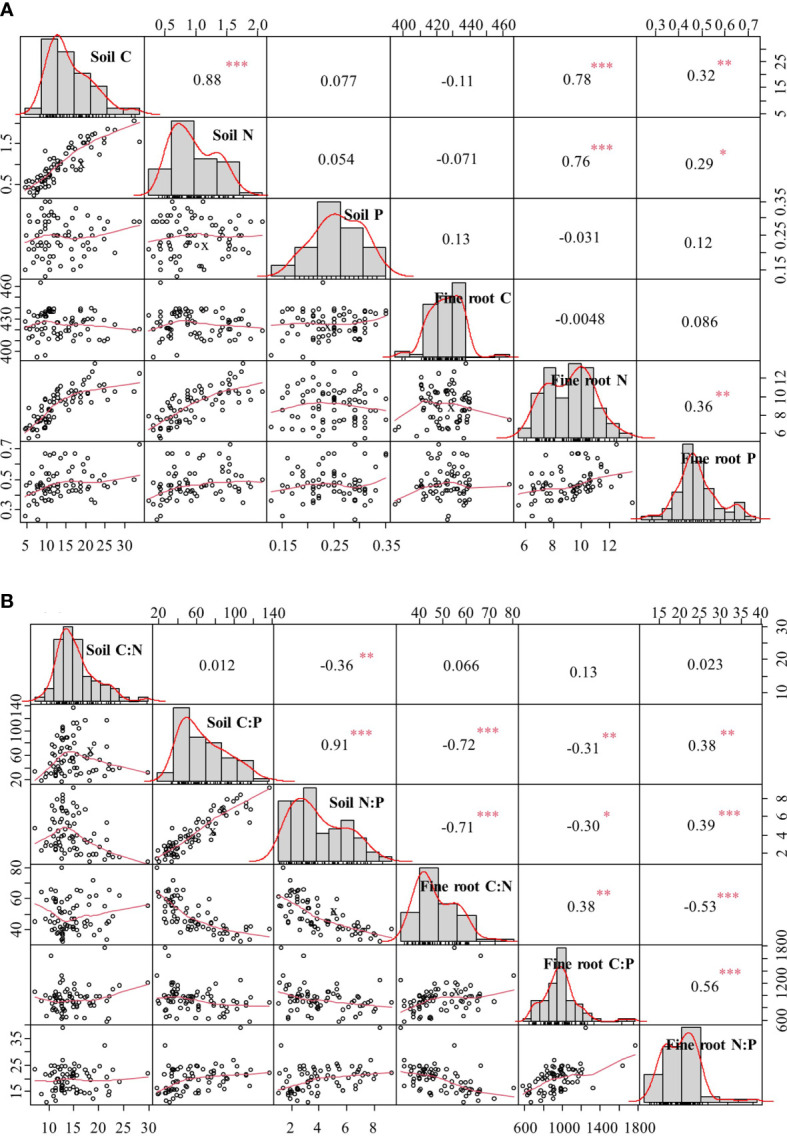
Correlation coefficients of C, N, and P contents **(A)** and C:N, C:P, and N:P ratios **(B)** in soil and fine roots. ^*^p < 0.05, ^**^p < 0.01, ^***^p < 0.001.

Significant positive correlations were found between SOC and soil TN, and between fine root N and fine root P (**Figure 4A**). Both SOC and soil TN showed significant positive correlations with fine root N and fine root P (**Figure 4A**). Soil N:P ratio was significantly positively correlated with soil C:P ratio but significantly negatively correlated with soil C:N ratio (**Figure 4B**). Both soil C:P and N:P ratios were significantly negatively correlated with fine root C:N and C:P ratios, but both were significantly positively correlated with fine root N:P ratio (**Figure 4B**). Moreover, fine root C:P ratio was significantly positively correlated to fine root C:N and N:P ratios, whereas fine root C:N ratio was significantly negatively correlated to fine root N:P ratio (**Figure 4B**).

## 4 Discussion

### 4.1 Soil C, N, and P contents and their stoichiometry during vegetation restoration

The average contents of SOC, TN, and TP at the topsoil (0–10 cm) in the four restoration stages in this study were 17.92, 1.32, and 0.24 g/kg^−1^ (**Figure 2**; **Supplementary Table S1**), respectively, which were lower than those in the average level in China (24.56, 1.88, and 0.38 g/kg^−1^, respectively; Tian et al., 2010). Previous studies have shown that soil C, N, and P contents decrease with decreasing latitude in forest ecosystems in China (Zhang et al., 2018; Chen X. et al., 2022). Therefore, the main reason for the relatively low soil C, N, and P contents could be the relatively low latitude of our study area (21.4°N). The C:P ratio (77.86, see **Supplementary Table S1**) and N:P ratio (5.72, see **Supplementary Table S1**) of topsoil in our study were higher than those in the average level in China (61 and 5.2, respectively; Tian et al., 2010), but they were more consistent with those of tropical soils on Hainan Island of southern China (79.73 and 5.41, respectively; Hui et al., 2021), which was related to the lower P content in tropical soils. The abundance of Fe/Al minerals in acidic soils results in higher weathering rates and increasing P fixation and leaching by P precipitation and adsorption (Vitousek et al., 2010); thus, soil in low latitude areas is generally P limited.

Our study showed that vegetation restoration significantly increased SOC and TN content, whereas TP content was not significantly affected (**Table 2**; **Figures 2A–C**). Natural restoration from grassland to forest results in a larger increase in vegetation productivity, litter production, and root biomass, and thus, a significant increase in both C and N returned to the soil (Xu et al., 2018; Wang and Zheng, 2021; Wu et al., 2022). Furthermore, the restoration of N-fixing tree species (e.g., the Fabaceae tree species *Archidendron eberhardtii* and *Ormosia pachycarpa*, see **Table 1**) also promotes the accumulation of N in the forest soil. Therefore, vegetation restoration could increase SOC and TN through multiple mechanisms such as litter and root turnover and biological N fixation in the study area. The findings from numerous studies are consistent with our results (Xu et al., 2018; Su et al., 2019; Wu et al., 2022). It has been suggested that the vegetation biomass increment leads to an increase in P uptake by plants and therefore a decrease in soil P content (Chen et al., 2000). However, other studies have suggested that increasing soil N stimulates roots to secrete more phosphatase, which promotes the breakdown of ester–phosphorus bonds in soil organic matter, thereby increasing soil P content (Wang et al., 2011). These inconsistent results may be due to the complex abiotic and biotic processes that determine soil P content (Vitousek et al., 2010). Soil P is primarily controlled by weathering and leaching and influenced by soil biogeochemical processes, soil parent material, and climate (Vitousek et al., 2010). In our sampling site, soil parent material, climate, and topography were highly similar among the restoration stages (see **Table 1**; **Figure 1**); therefore, the soil TP content remained relatively stable over time. This result is consistent with the recent finding of Lu et al. (2022), who also found that soil TP remains relatively constant along vegetation restoration in subtropical hills, southwestern China.

The soil C:N, C:P, and N:P ratios have been identified as indicators of soil quality and nutrient limitation in terrestrial ecosystems (Izquierdo et al., 2013; Bing et al., 2016). The C:N ratio reflects soil fertility and is used to assess the C and N balance in the soil (Tian et al., 2010). Similar to the results of Fan et al. (2015) and Ma et al. (2020), our results showed no significant effect of vegetation restoration on the soil C:N ratio (**Table 2**; **Figure 2D**). The constant C:N ratio in soils may be related to the close temporal coupling of C and N concentrations in the decomposition of litter (McGroddy et al., 2004; Yang and Luo, 2011). The soil C:P ratio is an indicator of soil P effectiveness and a measure of soil P immobilization by soil microorganisms (Tian et al., 2010), and soil N:P ratio is a predictor of N saturation and can be used to evaluate nutrient limitation thresholds (Peñuelas et al., 2012). In the present study, soil C:P and N:P ratios increased significantly with vegetation restoration (**Table 2**, **Figures 2E, F**), which is similar to the findings reported in many previous studies (Xu et al., 2018; Lu et al., 2022; Wu et al., 2022). SOC and TN content increased with vegetation restoration, but TP content did not change significantly (**Figures 2A–C**), leading to the increase in soil C:P and N:P ratios, confirming that the potential of soil microorganisms to release P from mineralized organic matter gradually decreases or remains stable over the different restoration stages. Therefore, vegetation growth is most likely affected by increasing P limitation in our study area.

We observed that SOC, TN, C:P, and N:P decreased with increasing soil depth except soil TP and C:N (**Table 2**; **Figure 2**), which is consistent with previous studies in tropical forests (Stone and Plante, 2014), shrublands, and grasslands (Wang et al., 2018; Yu et al., 2018). Litter decomposition process occurs mainly in the topsoil, thereby increasing the accumulation of topsoil nutrients (Xu et al., 2018). With increasing soil depth, the input of organic matter is limited due to the decrease in microbial decomposition activity and root uptake (Stone and Plante, 2014). Therefore, the SOC and TN contents gradually decreased from topsoil to subsoil in the study area. Because the weathering of rocks is extremely slow, the degree of weathering varies little in the 0–40-cm soil layer, resulting in no significant difference in TP content at different depths (**Figure 2C**). This finding is consistent with that by Su et al. (2019), who also found that soil TP content is independent of soil depth. Soil C:P and N:P ratios decreased with increasing depth (**Figures 2E, F**), which is also because the surface litter released more nutrients to the topsoil (Lilienfein et al., 2001; Bing et al., 2016).

### 4.2 Fine root C, N, and P contents and their stoichiometry during vegetation restoration

In the current study, the average C, N, and P contents of fine roots in the four restoration stages were 425.51, 9.09, and 0.47 g/kg^−1^, respectively, and the C:N, C:P, and N:P ratios were 48.87, 941.77, and 19.80, respectively (**Figure 3**; **Supplementary Table S1**). The average C, N, and P contents of fine roots of all Chinese plants were 473.9, 9.2, and 1.0 g/kg^−1^, respectively, whereas the C:N, C:P, and N:P ratios were 59.15, 522.10, and 14.27, respectively (Ma et al., 2015). We found that the C, N, and P contents and the C:N ratio of fine roots were lower, whereas the fine root C:P and N:P ratios were higher than those in all Chinese plants, which was attributed to the difference in climate, plant type, and soil nutrients in different regions (Zhang et al., 2018; Wang et al., 2020). Another study showed that the fine root C:N, C:P, and N:P ratios in tropical forests were 42.29, 1,035.72, and 22.09, respectively (Wang et al., 2020). These values are nearly identical to our study, indicating that the fine root C:N:P ratios may be well-constrained in the tropics.

We observed that vegetation restoration significantly increased fine root N and P contents, whereas fine root C content was almost unaffected (**Table 2**; **Figures 3A–C**). As a basic skeletal element of plants, C maintains good homeostasis in plants (Braakhekke and Hooftman, 1999). Similar to previous studies (Su et al., 2019; Su and Shangguan, 2021; Wu et al., 2022), our study also suggested that fine root C content was not affected by vegetation restoration. The increase in vegetation biomass during restoration caused plants to require more P- and N-rich substances (e.g., enzymes and transported proteins) to take part in metabolic activities, which increased the nutrient uptake by fine roots (Su et al., 2019). Furthermore, herbaceous plants have shallow roots, whereas shrubs and trees usually have deeper roots and are more capable of absorbing nutrients from different sources in the environment. Fine roots of graminoids (e.g., the dominant grass *Eragrostis Pilosa* and *Arundinella hirta*, see **Table 1**) have relatively low respiration rates and metabolic activity (Freschet et al., 2015; Roumet et al., 2016), resulting in reduced nutrient uptake rates in grassland. Moreover, Gao et al. (2019) confirmed that shrubs have stronger capacity than grasses to take up P from the soil. Recent findings based on meta-analysis revealed that plant type is the largest contributor to the shift in fine root C, N, and P contents and their ratios compared to soil and climate factors (Wang et al., 2020). Therefore, fine root N and P contents and their stoichiometric ratio in this study were most controlled by plant growth forms (herbs, shrubs, and trees) and soil features along the vegetation restoration gradient.

In the current study, the fine root C:N and C:P ratios significantly decreased, but N:P significantly increased with restoration (**Table 1**; **Figures 3D–F** ). The fine root C:N and C:P ratios indicate plant N and P utilization efficiency and the turnover rate of roots, and the higher ratios indicate a lower fine root turnover rate (Cao et al., 2020). Therefore, our study showed that vegetation restoration promoted the utilization of environmental N and P by plants, and the fine root turnover rate increased gradually. Previous studies have also shown that plant C:N and C:P ratios vary among vegetation types (Fan et al., 2015; Su et al., 2019). It is well known that N and P are the main factors affecting primary productivity and play a crucial role in plant metabolism, physiology, and growth (Güsewell, 2004). Therefore, the plant N:P ratio can characterize the availability of N and P in the soil. Previous studies suggested that plant N:P ratios <14 frequently signify N limitation, and N:P ratios >16 often suggest P limitation (Reich, 2005). In the present study, the average N:P ratio increased from 17.59 in grassland to 21.45 in primary forest, indicating that P limitation increased with vegetation restoration. Our results are similar to those of Huang et al. (2013) and Xu et al. (2018), who reported that the tropical vegetation in southern China is characterized by P limitation over time.

In this study, soil depth had a significant effect on fine root N content and C:N ratio (**Table 2**; **Figures 3B, D**). The differential response of fine root C and N contents to soil depth resulted in a decrease in the C:N ratio. Fine root nutrient contents and their stoichiometric characteristics are mainly influenced by the external biotic environment, especially soil nutrients at different depths and fine root morphology (Yuan and Chen, 2010). The finding of Makita et al. (2011) showed that fine root N content in topsoil (0–10 cm) was 1.4–2.5 times higher than in deep soil (40–50 cm), and the heterogeneity of soil nutrients may be the main factor contributing to the variation of fine root N content with soil depth. In this study, soil depth had no significant effect on fine root P content (**Table 2**; **Figure 3C**), which related to the fact that there was no significant difference in the vertical distribution of P in the soil profile (**Figure 2C**). In contrast, fine root C:N ratio increased with soil depth, which may be due to the decrease in soil N effectiveness with increasing depth (Hume et al., 2018).

### 4.3 Relationships between C:N:P stoichiometry in soil and fine roots

There were significant positive correlations between soil C and N contents and between these and fine root N and P contents (**Figure 4A**). The atmospheric CO_2_ is the source of plant C, and thus, soil C was an unlikely influence on plant C (e.g., fine root C). Therefore, there was indeed no significant correlation between soil and fine root C content (**Figure 4A**). The accumulation and decomposition of organic matter is the main source of soil N supply (Nardoto et al., 2014); thus, soil C content was significantly related to soil N content (**Figure 4A**). Fine roots are important organs for uptake and transport of soil nutrients, and their nutrient content is directly related to soil nutrient supply capacity (Makita et al., 2011). The accumulation and decomposition of soil organic matter release more available N, which promotes N and P uptake by fine roots with the restoration of vegetation. In addition, the increase in fine root biomass facilitates the release of root exudates and increases the efficacy of nutrients available to rhizosphere microorganisms, whereas the fine root turnover rate is accelerated, both of which contribute to the increase in SOC and TN. Therefore, fine root N and P contents can indicate soil fertility to some extent (Lambers et al., 2008).

In this study, both soil C:P and N:P ratios were significantly negatively related to fine root C:N and C:P ratios (**Figure 4B**). Soil C:P and N:P ratios increased with vegetation restoration (**Figure 2**), leading to more pronounced plant P limitation. Therefore, plants maintain normal physiological activities by increasing the amount of P uptake and resorption efficiency, which is one of the reasons for the increase in fine root P content and decrease in fine root C:P and N:P ratios. Both soil C:P and N:P ratios were significantly positively related to fine root N:P ratio (**Figure 4B**), implying that P fertilizer addition could reduce soil C:P and N:P ratios, thereby reducing fine root N:P ratio and alleviating the P limitation for vegetation in our study area (Mo et al., 2015; Van Langenhove et al., 2020).

Our study demonstrated the relationships between soil and fine root C, N, and P contents and their stoichiometric ratios during vegetation restoration, but these relationships may be modulated by soil extracellular enzymes (Sinsabaugh et al., 2008), soil microorganisms (Sun et al., 2020), and soil physicochemical properties (e.g., pH, moisture, and porosity) (Boudjabi and Chenchouni, 2022). Therefore, further studies are needed to analyze the association of these factors with soil and fine root ecological stoichiometric characteristics.

## 5 Conclusion

Overall, our study demonstrated that C, N, and P contents and their stoichiometry in both soil and fine roots responded significantly to vegetation restoration in a tropical mountainous area, southern China. Soil organic carbon, soil TN, and soil C:P and N:P ratios increased significantly with restoration and decreased significantly with increasing soil depth, whereas soil TP and C:N ratio were not significantly impacted. Moreover, vegetation restoration had no significant effect on fine root C content, but significantly increased fine root N and P content and N:P ratio and decreased fine root C:N and C:P ratios. Soil C:P and N:P ratios and fine root N:P ratio increased significantly during restoration, indicating that vegetation restoration increased P limitation of the ecosystem. Correlation analyses showed that both SOC and soil TN were significantly correlated with fine root N and fine root P, whereas soil C:P and N:P ratios were significantly correlated with fine root N:P ratio, indicating a reciprocal control of nutrient stoichiometric characteristics between soil and fine roots. Therefore, the application of an appropriate phosphorus fertilizer could reduce soil C:P and N:P ratios and fine root N:P ratio, consequently alleviating P limitation and promoting the restoration and resilience of degraded tropical forests. These results offer important insights into the restoration and sustainable management in tropical ecosystems.

## Data availability statement

The raw data supporting the conclusions of this article will be made available by the authors, without undue reservation.

## Author contributions

GH and LL: conception and design of the research. GH and ZZ: acquisition of data. GH: analysis and interpretation of data. GH and LL: statistical analysis. GH and LL: drafting the manuscript. GH, ZZ, and LL: revision of manuscript drafting and revision of manuscript. All authors contributed to the article and approved the submitted version.
