# Supplementary material for: Responses of carbon, nitrogen, and phosphorus contents and stoichiometry in soil and fine roots to natural vegetation restoration in a tropical mountainous area, Southern China
Source: Front Plant Sci. 2023 May 9;14:1181365. doi: 10.3389/fpls.2023.1181365 (PMC10203608; doi:10.3389/fpls.2023.1181365)
Supplement: Supplementary file 1 [file Table_1.docx]

Supplementary Material

# Supplementary Table

Table S1 The C, N, and P contents and their ratios in soil and fine roots at different depths. SE and CV mean standard error and coefficient of variance, respectively.

| Soil depth (cm) |  | Soil | | | | | |  | Fine root | | | | | |
| --- | --- | --- | --- | --- | --- | --- | --- | --- | --- | --- | --- | --- | --- | --- |
|  |  | C | N | P | C:N | C:P | N:P |  | C | N | P | C:N | C:P | N:P |
| 0-10 | Mean | 17.92 | 1.31 | 0.24 | 13.92 | 77.86 | 5.72 |  | 426.99 | 10.02 | 0.48 | 44.29 | 910.31 | 21.21 |
|  | SE | 1.44 | 0.09 | 0.01 | 0.62 | 5.92 | 0.41 |  | 2.72 | 0.39 | 0.02 | 1.92 | 32.79 | 1.07 |
|  | SV | 38.55 | 34.26 | 22.74 | 21.39 | 36.49 | 34.45 |  | 3.06 | 18.66 | 18.27 | 20.83 | 17.27 | 24.27 |
| 10-20 | Mean | 13.90 | 1.02 | 0.25 | 13.92 | 55.65 | 4.08 |  | 425.13 | 8.84 | 0.48 | 49.95 | 934.02 | 19.05 |
|  | SE | 1.25 | 0.08 | 0.01 | 0.80 | 5.08 | 0.34 |  | 2.25 | 0.35 | 0.02 | 2.03 | 44.88 | 0.84 |
|  | SV | 43.17 | 37.63 | 18.97 | 27.51 | 43.80 | 40.38 |  | 2.54 | 19.03 | 21.63 | 19.53 | 23.04 | 21.16 |
| 20-40 | Mean | 9.51 | 0.62 | 0.25 | 16.35 | 41.18 | 2.68 |  | 424.41 | 8.41 | 0.45 | 52.35 | 980.99 | 19.15 |
|  | SE | 0.61 | 0.05 | 0.01 | 0.98 | 4.02 | 0.29 |  | 2.02 | 0.32 | 0.02 | 2.23 | 47.96 | 0.94 |
|  | SV | 30.86 | 37.96 | 21.71 | 28.76 | 46.76 | 51.46 |  | 2.29 | 18.28 | 21.15 | 20.48 | 23.45 | 23.46 |
| 0-40 | Mean | 13.78 | 0.98 | 0.25 | 14.73 | 58.23 | 4.16 |  | 425.51 | 9.09 | 0.47 | 48.87 | 941.77 | 19.80 |
|  | SE | 0.77 | 0.06 | 0.01 | 0.48 | 3.39 | 0.25 |  | 1.34 | 0.22 | 0.01 | 1.24 | 24.37 | 0.56 |
|  | SV | 47.38 | 47.17 | 21.33 | 27.60 | 49.12 | 50.27 |  | 2.66 | 20.15 | 20.56 | 21.44 | 21.81 | 23.67 |
